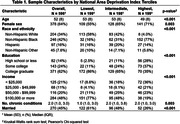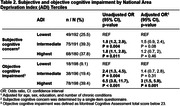# Neighborhood Deprivation, Subjective and Objective Cognitive Impairment

**DOI:** 10.1002/alz.089352

**Published:** 2025-01-09

**Authors:** Britney Sun, Fangyu Yeh, Pauline Zheng, Laura M Curtis, Julia Yoshino‐Benavente, Han Q Luu, Patrick Cecil, Prophecy Agyare, Rodolfo Zuleta, Michael S Wolf, Minjee Kim

**Affiliations:** ^1^ Northwestern University, Chicago, IL USA; ^2^ Northwestern University Feinberg School of Medicine, Chicago, IL USA

## Abstract

**Background:**

Social determinants of health have been associated with disparities in health outcomes, including cognitive impairment and Alzheimer’s disease. While individual‐level disparities have been characterized, more research is needed into structural social determinants of health (SSDoH) and their association with cognitive outcomes, especially for those in midlife. This study aimed to investigate the association between SSDoH and subjective and objective measures of cognitive impairment.

**Methods:**

English‐speaking adults ages 35‐64 were recruited from an academic general internal medicine practice and federally qualified health network in the Chicagoland area. SSDoH was evaluated using the Area Deprivation Index (ADI), a validated factor‐based index of neighborhood socioeconomic context using participant census block, then divided into terciles with the highest tercile indicating highest neighborhood deprivation. Subjective cognitive concern was measured by a single question assessing concern about cognitive or memory issues as part of the Everyday Cognition Cog scales. Objective cognition was measured using the Montreal Cognitive Assessment (MoCA) with MoCA < 23 (out of 30) indicating cognitive impairment. We used univariate and multivariable logistic regression models to characterize the relationship between SSDoH and cognitive impairment, including *a priori* covariates of age, sex, education, and number of chronic conditions.

**Results:**

A total of 596 participants (age 52±8; 64% female; 42% non‐Hispanic Black, 34% non‐Hispanic White, 16% Hispanic; 62% college graduate) were included in the analysis. Younger age, female sex, non‐Hispanic Black race, and lower education and income were associated with higher ADI (**Table 1**). One‐third of participants (34%) reported subjective cognitive concern, whereas objective cognitive impairment was found in 22.6%. Compared to the lowest ADI tercile (i.e. least deprived), the highest ADI tercile (i.e. most deprived) was significantly associated with objective cognitive impairment (aOR, 3.0; 95% CI, 1.5‐5.9; p = 0.001) but not subjective cognitive concerns (aOR, 1.2; 95% CI, 0.7‐2.0; p = 0.46), after adjusting for *a priori* covariates (**Table 2**).

**Conclusions:**

Neighborhood deprivation is associated with Future research should investigate the possible mechanisms of this association to identify optimal interventions at the neighborhood level.